# Novel hybrid method to additively manufacture denser graphite structures using Binder Jetting

**DOI:** 10.1038/s41598-021-81861-w

**Published:** 2021-01-28

**Authors:** Vladimir Popov, Alexander Fleisher, Gary Muller-Kamskii, Shaul Avraham, Andrei Shishkin, Alexander Katz-Demyanetz, Nahum Travitzky, Yair Yacobi, Saurav Goel

**Affiliations:** 1 Israel Institute of Metals, Technion, Israel Institute of Technology, 3200003 Haifa, Israel; 2 Institute of New Materials and Technologies, Ural Federal University, Ekaterinburg, Russia; 3 NRCN, Nuclear Research Center Negev, 8419001 P.O.Box 9001, Beer-Sheva, Israel; 4 Rudolfs Cimdins Riga Biomaterials Innovations and Development Centre of RTU, Institute of General Chemical Engineering, Faculty of Materials Science and Applied Chemistry, Riga Technical University, Riga, 1007 Latvia; 5 Maritime Transport Department, Latvian Maritime Academy, Riga, 1016 Latvia; 6 Department of Materials Science and Engineering, Institute of Glass and Ceramics, University of Erlangen-Nuremberg, 91058 Erlangen, Germany; 7 School of Engineering, London South Bank University, London, SE10AA UK; 8 School of Aerospace, Transport and Manufacturing, Cranfield University, Cranfield, MK43 0AL UK; 9 Department of Mechanical Engineering, Shiv Nadar University, Gautam Budh Nagar, 201314 India

**Keywords:** Ceramics, Characterization and analytical techniques, Design, synthesis and processing, Mechanical engineering

## Abstract

This study introduces two hybrid processes integrating an additive manufacturing technique with post-processing treatments namely (i) Binder Jetting Printing (BJP) + Cold Isostatic Pressing (CIP) + cycle and (ii) BJP + cycle where cycle refers to a sequence of Impregnation—Drying—Pyrolysis. These two new processes yielded additively manufactured parts with higher density and reduced defects/porosities. As a testbed, we used these new processes to fabricate graphite structures. The samples produced by both methods were compared with each other and benchmarked to the samples produced by (a) BJP alone and (b) Traditional uniaxial pressing like compaction moulding. Various characterisation methods were used to investigate the microstructure and mechanical properties which showed that the porosity of hybrid manufactured samples reduces from 55% to a record 7%. This technological pathway is expected to create a new avalanche of industrial applications that are hitherto unexplored in the arena of hybrid additive manufacturing with BJP method.

## Introduction

Graphite is a widely used material in various industrial applications due to its thermal stability at elevated temperatures, chemical resistance to aggressive aqueous solutions and high electrical conductivity^1^. Some of the common industrial examples of this includes manufacturing of melting crucibles, heating elements as well as in the processing of the bow of ballistic missiles and spacecraft for thermal protection^2^ and for the production of various elements and tools of electric machines (brushes), electric vehicles and pumping equipment (e.g. blades)^3^.

Mechanically stable graphite products are traditionally produced using the powder metallurgy technique which relies on using graphite powder and a binder. The binder is a “glue” that bonds the powder's particles together. In traditional manufacturing, upon heating (e.g. coal resin or petroleum pitch) the binder softens, allowing a uniform wetting and homogenous adhesion of the graphite powder. The resulting blend after milling or grinding moves to the next production steps such as moulding, compaction or extrusion.

Polymer-bonded graphite is popularly used for tribological applications. These are used in high production volumes and thus, they turn out to be low cost and can easily be produced in complex desirable shapes^4^.

**Figure 1:**
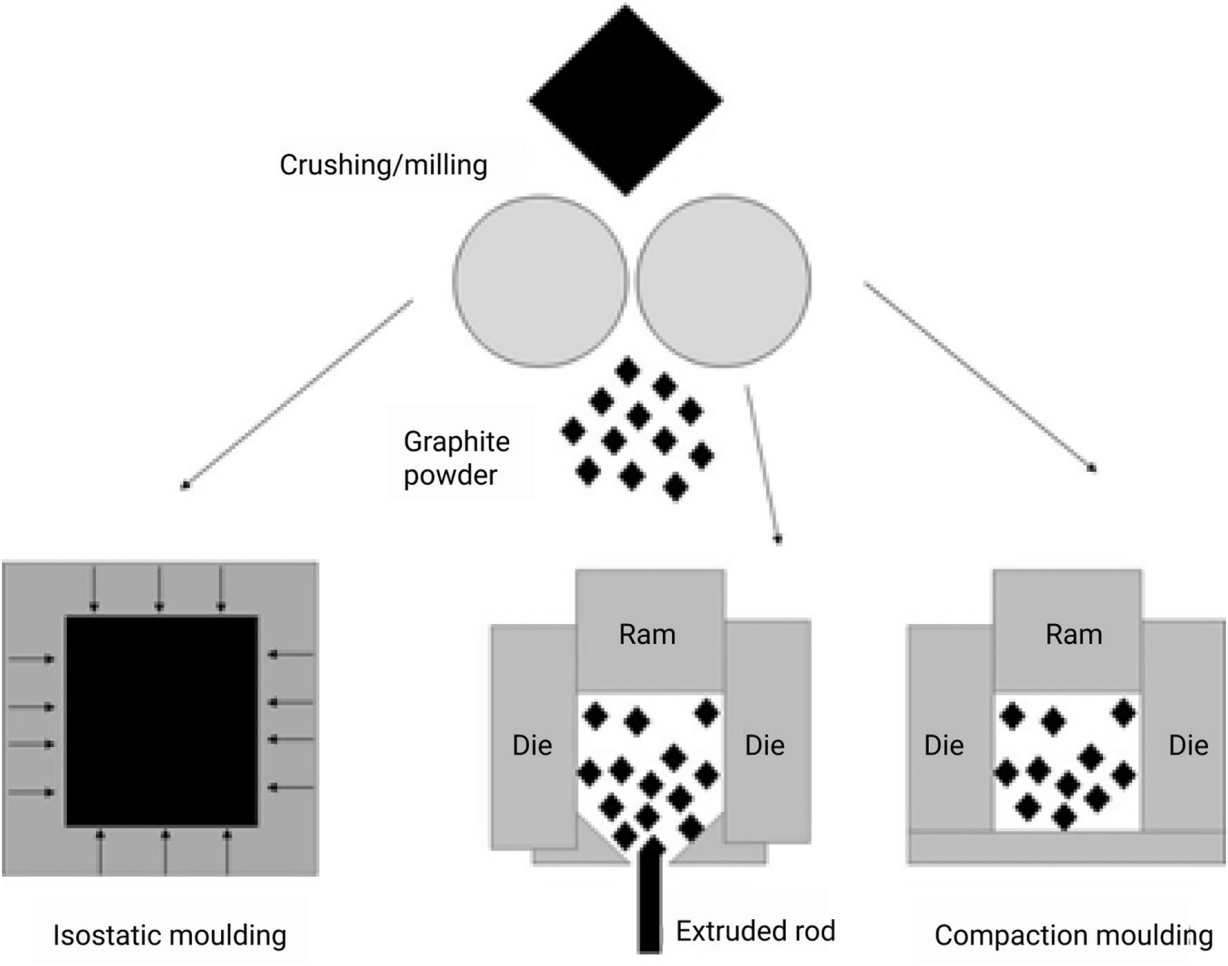
Production techniques for graphite structures (left) isostatic pressing or moulding (middle) extrusion and (right) uniaxial compaction.

The three main production techniques (see Fig. 1) for graphite structures having different final material properties are (i) isostatic pressing (moulding)—where graphite powder mixture is pressed isostatically (ii) extrusion—where graphite powder mixture is pushed through a die and (iii) uniaxial compaction (moulding) where graphite powder is pressed in one direction (e.g. for manufacturing graphite cylinders)^5^.

The control over the density of graphite components is crucial, since density plays a vital role in influencing the strength and many other functional characteristics of the component. Insufficient density of the green-bodies may cause post-processing troubles, reduced strength and low wear resistance^6–8^.

Usually carbon-based materials are used as reinforcements in additively manufactured metallic parts^9–11^ and composites^12,13^. Most of the conventional components of carbon and its derivatives are made through traditional subtractive manufacturing processes. Owing to the inability of current subtractive technologies to produce complex-shaped graphite parts with desired microstructures and properties, novel additive processing techniques, such as Selective Laser Sintering (SLS), Stereolithography (SLA), Laminated Object Manufacturing (LOM) and Binder Jetting Printing (BJP) have been seen to emerge during the last two decades. These techniques offers capability to fabricate ceramic bodies with complex geometry^14–18^.

The stereolithography method uses laser/light projection and photopolymerization processes for fabricating multimaterial heterogeneous structure^9,10^. The thermoset material is utilised as a matrix for powder reinforced materials. Reported data shows that carbon-containing composites could be manufactured using graphite powder, graphene, and carbon nanotubes by SLA-like 3D printing^10^.

Another technique, LOM is used for papers, polymers and metals. The LOM process can be used to produce ceramic (e.g. Al_2_O_3_, Si_3_N_4_, and SiC with graphitic powder/carbon fibers) papers^11^.

SLS enables additive manufacturing of carbon-containing structures by working with material mixing. For example, the SiC powder, short carbon fibers and epoxy resin are ball-milled and then processed by SLS. The printed parts can be carbonized and then infiltrated with molten silicon to obtain reaction bonded SiC structures^12^. Other Powder Bed Fusion (PBF) techniques, like Selective Laser Melting (SLM) and Electron Beam Melting (EBM) cannot be used for ceramics manufacturing. These techniques are applied to metals, and in some cases to reinforced alloys and metal matrix composites^13–16^.

The unique benefit of BJP in comparison to the Powder Bed Fusion techniques is its capability to process virtually any powder including graphite, ceramics and refractory alloys^17^. Here, the binder can be removed from the printed green part by drying which then leaves behind an unbonded green porous part^18–21^.

Attempts have recently been made to apply BJP to manufacture graphite-based structures with required physical properties^18–20,22,23^. One of the advantages of the BJP process is the capability to achieve the required density and mechanical performance using typical post-processing such as the reaction sintering or liquid metal infiltration^22,24,25^. The limited reported data about the possible density of graphite and carbon-containing materials by BJP shows for instance that the part density of graphene-based electrodes processed by BJP was only 0.44 g/cm^3^^19^. To the best of author’s knowledge, there is no other report about printing of high-density graphite produced by the BJP method. The low flowability of irregular-shape graphite powders limits their effective use for additive manufacturing since freshly printed structures have a large number of defects after powder deposition^23^.

The production-related porosity induced in the graphite materials (both in subtractive and additive manufacturing) results in a certain permeability to fluids which limits the use of such materials in applications i.e. sealing elements.

In some cases, open porosity is required for reaction bonding and liquid infiltration treatment, which can radically increase the density^18,22,25,26^. In order to reduce the porosity of the fabricated samples, and to increase the density of the final material, post impregnation can be used^4,18,22^.

Overall, BJP is clearly the most promising technique for fabricating carbon-based and graphite parts due to the possibility of incorporating intelligent functions like heating and thermal management. Also, the components manufactured using BJP can be imbued with specific properties of graphite and carbon which includes mechanical strength, light-weight, higher electrical and thermal conductivity and low thermal expansion^21^.

The present work was aimed at investigating the feasibility of producing additively manufactured low-porosity/high-density graphite parts using the BJP technique combined with technologically necessary post treatments, namely binder impregnation and pyrolysis. Cold isostatic pressing (CIP) was employed as an intermediate density improvement method as an interim processing stage. The main research hypothesis in this research was that there lies a tradeoff between the achievable density and the ease with which complex shapes can be fabricated so a hybrid method can balance these requirements. The combination of techniques can lead to lower porosity and improved mechanical and physical properties of complicated-shaped manufactured parts^27,28^. Determination of process critical steps and the parameters critical for achieving the density improvement were found which are being reported in this work.

## Methods and materials

### Experimental scheme

**Figure 2:**
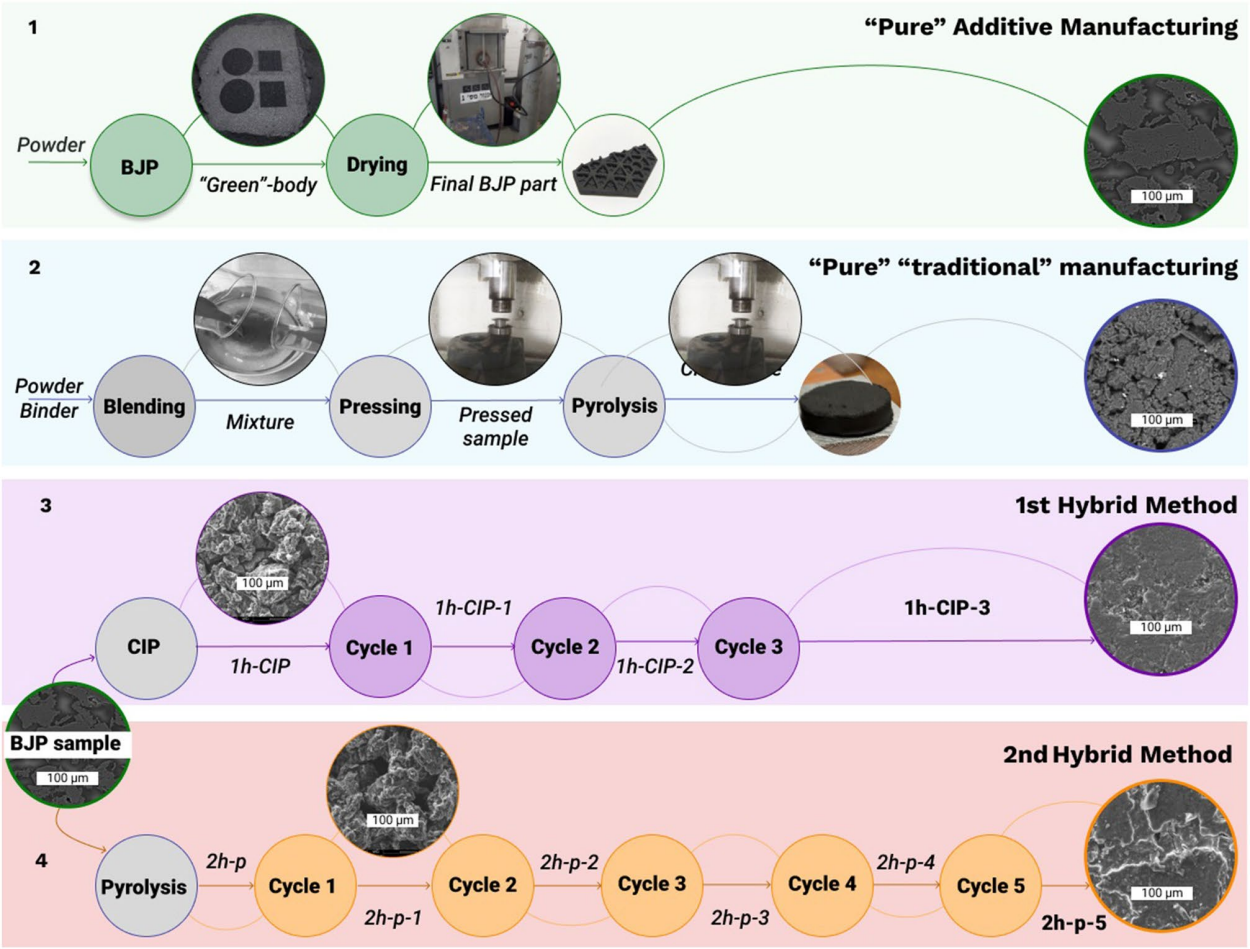
Process flow showing production of graphite using different production routes where *BJP* Binder Jetting Printing, *CM* compacted moulding, *CIP* Cold Isostatic Pressing, Cycle is the sequence of Impregnation—Drying—Pyrolysis.

Figure 2 illustrates the processing routes used in this work. The first line in Fig. 2 is devoted to the “purely” additive manufacturing stage using the BJP technique alone. As was explained above, such a method enables production of complex geometrical structures, however the porosity of such parts is high (~ 50–60%), and their density is low^22,24,29^. The second route involved uniaxial pressing (compaction moulding) and was performed purely to illustrate the density/porosity achievable from the “traditional” route (CM-samples). In the third and fourth lines, we illustrate two new methods starting from BJP and then using secondary techniques of treatments. The BJP processed part can either be CIPed after printing and then passed through three cycles of binder impregnation and pyrolysis (1 h-samples—1st hybrid method) or can be routed via five cycles of pyrolysis and impregnation (2 h-samples—2nd hybrid method). Overall, the objective was to combine the advantages of AM (freedom of design) and traditional manufacturing (high-density). As a testbed, we fabricated a complex-shaped part which was processed further by the 2nd hybrid method. This part was in the form of a 50 mm-side hexagonal mirror. The height of the inner ribs was 4 mm, their thickness was 1.5 mm. The thickness of the outer plane was 2.5 mm.

Through the manufacturing experiments, a total of ten rectangular prism-shaped samples (18 × 14.5 × 6 mm^3^) were printed for the microstructural analysis and mechanical testing. We observed that the geometry of the part has no influence on the density of the green parts^30,31^. The rectangular prism shape for BJP samples were selected for further characterization. The CM samples had a cylindrical shape (d = 30 mm, h = 7 mm) due to the moulding die dimensions. The hybrid manufactured 1 h- and 2 h-samples initially had the same size and prism bar-shape as BJP ones since the new processes start after the BJP process.

### Binder Jetting Printing (BJP)

**Figure 3:**
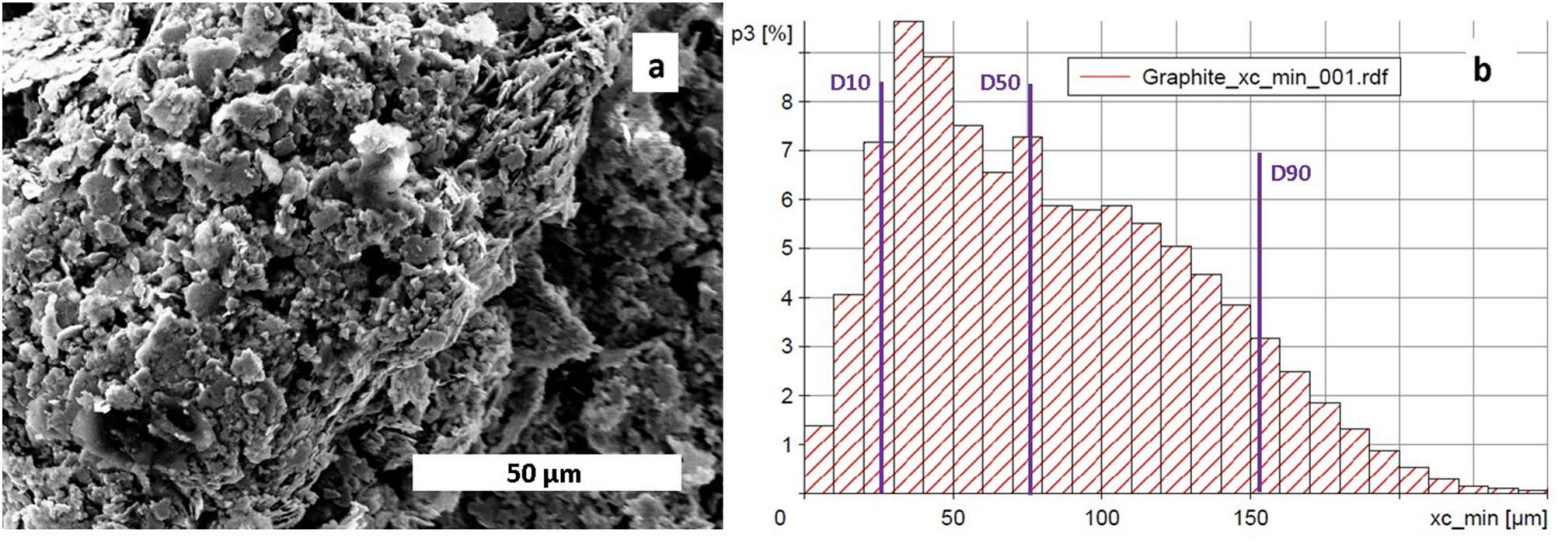
(**a**) Scanning electron microscopy (SEM) image showing graphite powder morphology and (**b**) Particle size distribution of the graphite powder used.

Specimens for the current study were manufactured using BJP *ExOne M-Flex* machine (*ExOne*, USA). The graphite powder with purity of > 98% and an average particle size of 80 µm was used. The morphology of the powder and particle size distribution obtained prior to the study are shown in Fig. 3a,b. The reason for use of BJP was due to the advantages of sample shaping. Unlike other methods, the BJP method does not require a rigid die for sample preparation^32^. Moreover, BJP can produce geometrically complex parts free from residual stresses and with controlled porosity^24^.

In carrying out the BJP, a commercial *ExOne* phenolic binder was used, this was because the phenolic binder after debinding leaves residual carbon in as print specimen^22^. The binder density according to technical documentation was ρ_b_ = 0.94 g/cm^3^. The BJP parameters used were layer thickness of 100 μm, powder bed temperature of about 44 °C and layer drying speed of 25 mm s^−1^.

The BJP printing settings include binder saturation *S—*a quantitative parameter of binder amount in pores. For the experiments, *S* was 60%. This means that after the BJP process, 60% of pores were filled by the binder while 40% were empty voids. The powder packing rate was 60%. The volume of binder in printed structures can be calculated using the following equation:

1 $$S = V_{b} / \, V_{p} ,$$

where *V*_*b*_ stands for volume of binder and *V*_*p*_ for pores’ volume^33^.

For example, for a 1 cm^3^ cube sample: *V*_*p*_ = 40% = 0.4 cm^3^; *V*_*b*_ = 0.4 × 0.6 = 0.24 cm^3^; layer thickness 100 μm; binder mass for the sample is *m*_*b*_ = *V*_*b*_ × ρ_b_ = 0.226 g; the mass of binder in one layer *m*_*b/l*_ = 2.26 mg/cm^2^.

Layer thickness for all experiments was maintained to be the same i.e. 100 μm. This parameter is flexible and can be changed depending on product requirements. The machine’s software uses layer thickness value for automatic calculation of the binder saturation.

The printed envelope volume *V*_*PE*_ is defined as:

2 $$V_{PE} = \, X_{d} \times \, Y_{d} \times {\text{ layer}}\,{\text{thickness }}\left( {Z_{d} } \right)^{{{31}}} ,$$

where *X*_*d*_ and *Y*_*d*_ are the corresponding distances between successive droplets in X and Y directions respectively. *Z*_*d*_ is the current building height. From (2), a direct relation between the layer thickness and the binder saturation is evident and this is the reason behind "green" body’s mechanical properties and dimensional accuracy^31^.

### Drying, impregnation and pyrolysis

After BJP, the samples were dried in the furnace for 2 h at 200 °C. It has been shown that the density of printed preforms can be increased by impregnation of phenolic resin^4,34^. Phenolic binder is advantageous for this purpose because the residual carbon after debinding (pyrolysis) partially fills the pores. This treatment is called Phenolic Resin Binder Impregnation (PRBI)^22^. In this work, all impregnations were carried out using the same phenolic binder that was used for the Binder Jetting Printing.

For impregnation, the BJP samples after drying were placed in the binder at room temperature for 1 h under reduced pressure.

Before pyrolysis, the impregnated samples were dried under the same conditions i.e. 2 h at 200 °C in electric muffle Nabertherm furnace (Germany). The pyrolysis was carried out using argon at 1000 °C for an hour.

### Cold isostatic pressing (CIP)

Before CIP treatment, the printed graphite samples were vacuum packed in polyethylene bags. They were then isostatically pressed at 106 MPa for 1 min at room temperature.

### Compaction moulding

To compare the possible densification of the BJP-made samples, additional samples were manufactured by compaction moulding with the same phenolic binder. An experimental ratio of binder amount to powder of 1:3 was used corresponding to the amount of binder (*V*_*b*_ = 24%) in the BJP process. The mixture was prepared using a blending machine. It was uniaxially pressed using a laboratory press for 20 min under 20 MPa.

### Microstructure and phase composition analysis

The samples were prepared using epoxy mounting and standard polishing with abrasive papers and SiO_2_ finishing.

The scanning electron microscopy analysis was performed by SEM FEI Inspect (FEI, Brno, Czech Republic) equipped with Electron Probe Micro Analyzer (EPMA). A Back-Scattered Electrons Detector was used to obtain phase contrast. The acceleration voltage and working distance used were 20 kV and 9–11 mm, respectively.

A Nikon Eclipse LV150 (Japan) microscope was used for the optical analysis. Image analysis was conducted using Olympus Stream Essentials software.

X-rays diffraction (XRD) was employed to examine the phase content of as built vs the impregnated samples. Stationary Rigaku Smart Lab diffractometer (Tokyo, Japan) equipped with a Cu tube (λ_Kα_ = 1.5406 Å) was used. The scattering range (2θ) was 5 ÷ 50°.

Microhardness HV10 evaluation was performed using a Vickers Hardness Tester FV-110 as discussed elsewhere^35^.

## Results and discussions

The two main characteristics of the fabricated parts to be evaluated from the additional analysis made were porosity and density and the mechanical properties, these are discussed next.

### Porosity and density of the parts

**Figure 4:**
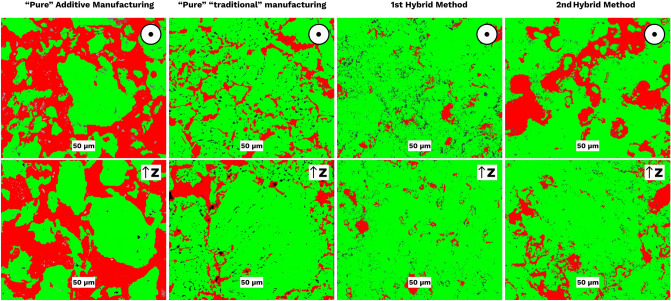
Image analysis of optical microscopy images of crosscuts in XY- and Z-planes. The black arrow points at the build/pressing direction (Z), i.e., perpendicular to the layers; the black circle with the point in the center corresponds to XY plane–parallel to layers.

Figure 4 shows a comparison of the porosity in (i) the green-body after pure additive manufacturing (BJP alone), (ii) uniaxially pressed sample (CM), (iii) BJP + CIP and 3 impregnation cycles (1st hybrid) and (iv) BJP + pyrolysis + 5 impregnation cycles (2nd hybrid).

Figure 4 shows the influence of the treatments revealing the variation in the porosity of the fabricated samples. It can be observed that the 1st hybrid sample showed reduced volume fraction of porosity compared to all other methods. It can be observed that in the “green” printed samples, the porosity level was comparable to one with the freely poured powder (correspondingly BJP and S0 samples in Table 1). As shown in Fig. 4, in the 1st hybrid method, the dense phase formed homogeneously in both directions. Further calculations shown in Table 1 confirmed this aspect quantitatively. In Table 1 are presented the porosity level (column Pores, %) and the percentage of the dense part (column Powder, %). The column "Powder, %" shows that the 2nd hybrid method provides a similar density as compaction moulding. Moreover, the 1st hybrid method demonstrates even higher density. The values shown here are averaged using 3 images in each plane.

**Table 1:** Image analysis showing volume fraction of porosity “green”, under treated, and isostatically pressed samples.

Sample	Plane	Volume fraction of			
		Pores, %	Standard deviation	Powder, %	Standard deviation
S0 (powder)	–	59.82	2.45	36.13	2.56
BJP (“green”-body)—Pure Additive	z	48.98	0.74	49.13	1.13
	xy	56.24	2.05	40.67	2.12
CM (compaction moulded)—Traditional	z	15.21	1.53	81.12	1.78
	xy	15.69	1.52	80.33	1.92
1 h-CIP-3 (1st hybrid)	z	7.32	1.64	88.72	2.20
	xy	7.34	1.20	90.83	1.97
2 h-p-5 (2nd hybrid)	z	18.91	1.27	79.13	1.6
	xy	18.76	1.78	77.61	2.40

The initial porosity level achieved from the BJP made green-body samples (S1) was higher than 50%. The 1st hybrid method with post CIPing lead to the highest densification of the printed samples with an average porosity of 7%. The significant pore closure resulted in the shrinkage which is typical in ceramic manufacturing. For BJP made graphite, volumetric shrinkage of about 20% was observed. The 2nd hybrid method with the use of impregnation and pyrolysis cyclic route decreases the porosity up to 18%.

Since the starting density is similar in the BJP and hybrid starting samples, the density evolution rate is dependent on the pyrolyzed residual graphite mass added in each cycle. However, it should be taken into account that after a certain number of cycles of phenolic reaction binder impregnation (PRBI) and pyrolysis, the density improvement is limited because of the formation of a dense layer near the surface of the sample that prevents further carbonization^36^.

**Figure 5:**
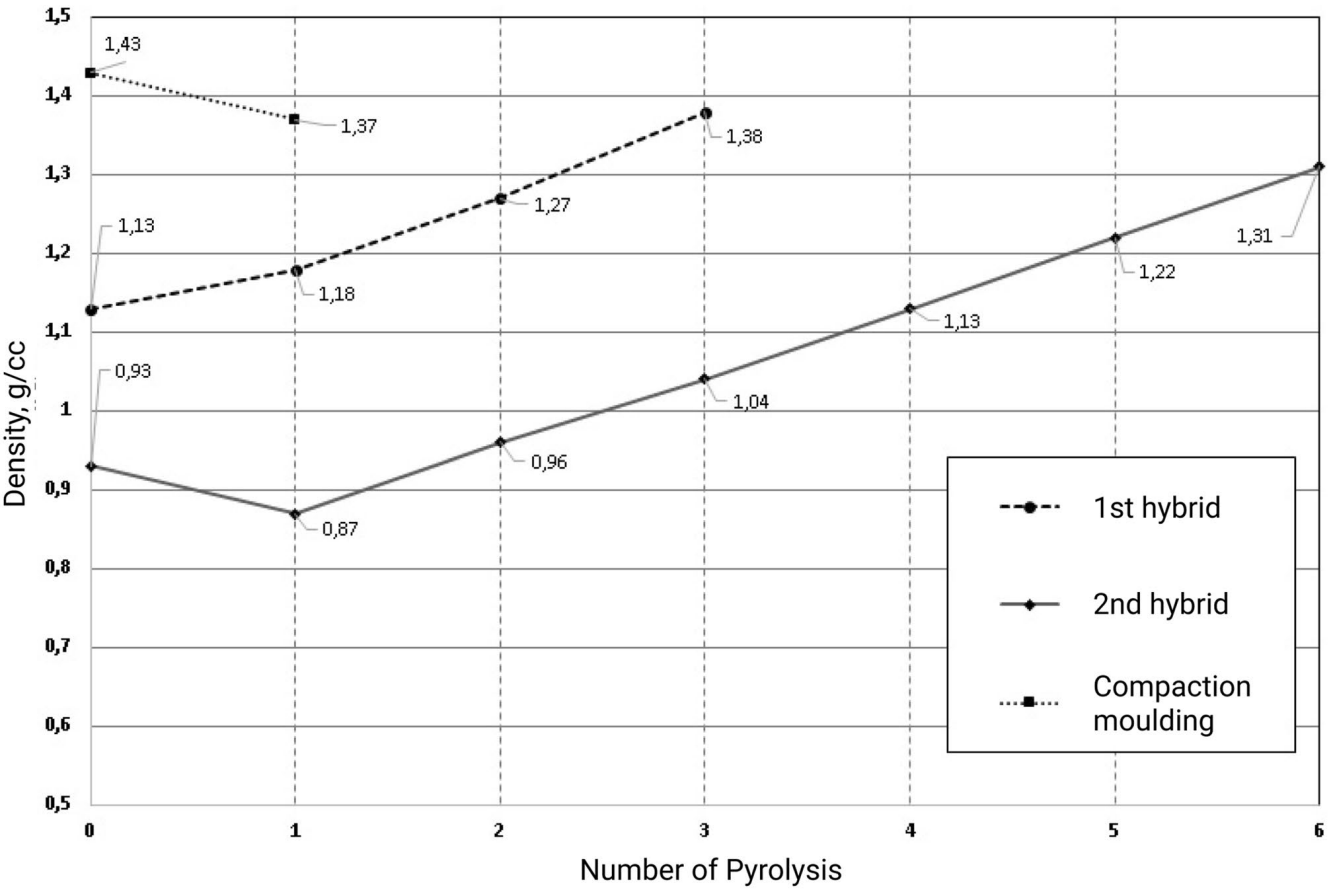
Dependence of the hybrid and compacted samples density on the number of pyrolysis cycles. 2nd to 6th pyrolysis was applied after PRBI. For compacted sample as initial density is the density before pyrolysis.

As shown in Fig. 5, the hybrid graphite structures became denser with the successive PRBI and pyrolysis cycles. The density was observed to depend on the fabrication method and on the number of cycles. The proximity in the value of density was confirmed by calculations (see Table 2, Fig. 5).

**Table 2:** Graphite samples prepared according to each step of the process flow.

Sample	State	Size (mm)	Weight (g)	Density (g/cm^3^)
S0	Powder	–	–	0.62
BJP	Green-body	18 × 14.5 × 6	1.47	0.94
CM	Compaction moulding and pyrolysis	d = 30; h = 7	6.9	1.37
1 h-CIP	BJP “green”-body after CIP	16 × 14 × 5.6	1.42	1.13
1 h-CIP-1	CIPed after Cycle 1	16 × 14 × 5.6	1.49	1.18
1 h-CIP-2	CIPed after Cycle 2	16 × 14 × 5.6	1.60	1.27
1 h-CIP-3	CIPed after Cycle 3	16 × 14 × 5.6	1.73	1.38
2 h-p	BJP “green”-body after 1st pyrolysis	18 × 14.5 × 6	1.37	0.87
2 h-p-1	After Cycle 1	18 × 14.5 × 6	1.51	0.96
2 h-p-2	After Cycle 2	18 × 14.5 × 6	1.63	1.04
2 h-p-3	After Cycle 3	18 × 14.5 × 6	1.78	1.13
2 h-p-4	After Cycle 4	18 × 14.5 × 6	1.91	1.22
2 h-p-5	After Cycle 5	18 × 14.5 × 6	2.05	1.31

The density of samples was calculated through weight measurement (see Table 2, Fig. 5). As can be seen from Table 2 and Fig. 5, the 2nd hybrid sample had 96% bulk density compared to compaction moulded sample (CM). The 1st hybrid sample that passed CIP had even higher density—1.38 g/cm^3^ accounting for 100% density of the reference sample (CM).

One key drawbacks of the above-mentioned BJP process in the production of graphite-based structures is the large amount of residual porosity, a possible method of improvement is offered by CIP before the binder impregnation cycles.

**Figure 6:**
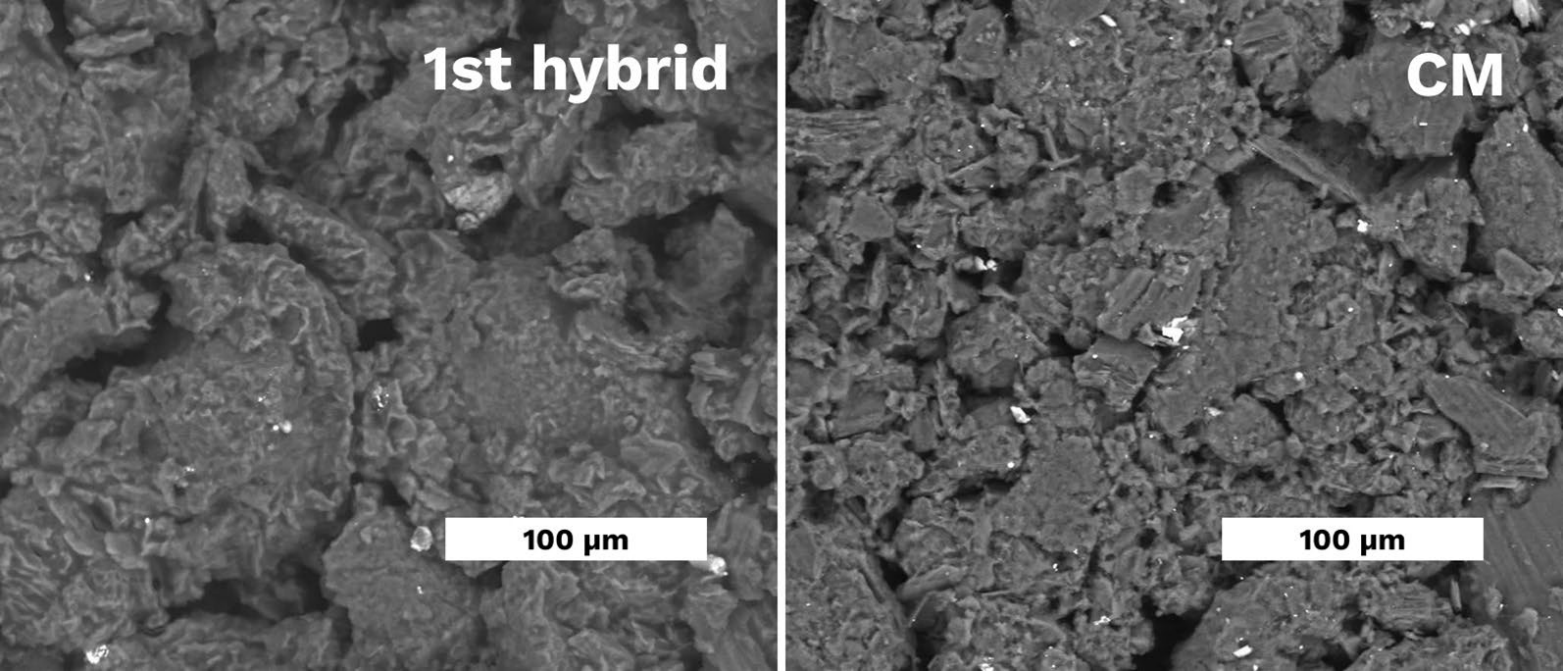
SEM images of the 1st hybrid sample (1 h-CIP-5) and *CM* compaction moulded sample.

Figure 6 shows the degree of similarity between the microstructure of the samples fabricated by BJP with post-CIPing and resin impregnation (1st hybrid) and compaction moulding. That corresponds to density measurements in Fig. 5.

### XRD examination

**Figure 7:**
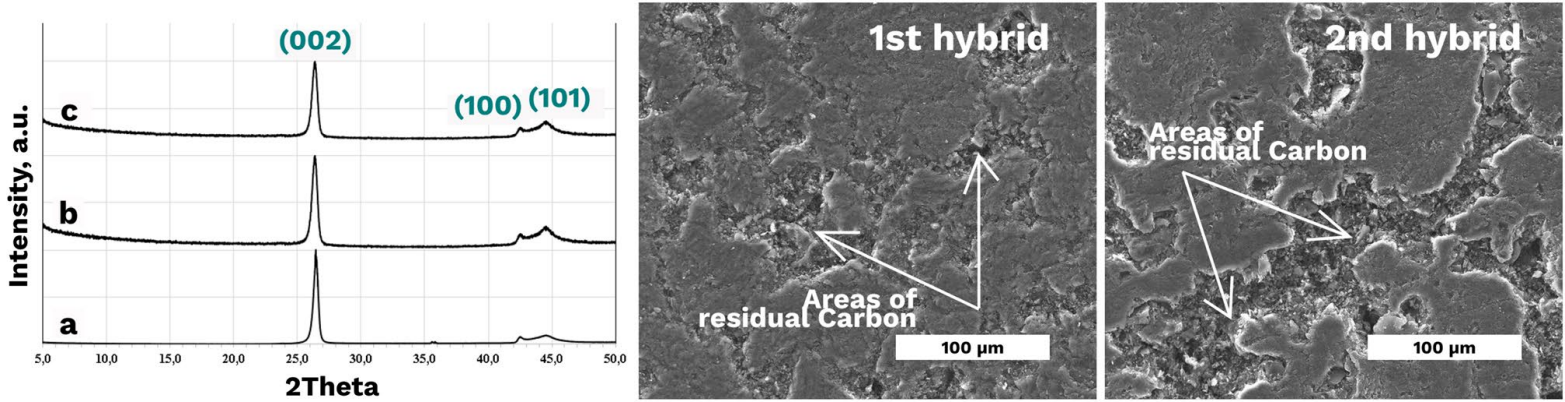
XRD spectra of the: (**a**′) BJP; (**a**″) 2 h-p-5 sample; (**a**‴) 1 h-CIP-3 sample; (**b**,**c**) SEM images of the 1st hybrid sample (1 h-CIP-3) and 2nd hybrid sample (2 h-p-5).

The XRD examination of the as-built (BJP) and highly impregnated 1st and 2nd hybrid specimens (Fig. 7a) did not reveal any new crystalline and amorphous phases aroused from impregnation. Therefore, the impregnation-induced graphite phase had a crystallographic structure corresponding to that of the matrix graphite phase.

The samples CM, 1 h-CIP-3 and 2 h-p-5 were final samples from each of the following manufacturing routes: compaction moulding (CM), 1st hybrid method (BJP after CIP and three cycles of PRBI and pyrolysis), and 2nd hybrid method (BJP after 5 cycles of PRBI and pyrolysis).

From Fig. 7, it can be observed visually that the initial porosity of the 2nd hybrid sample 2 h-p-5 still remains. However, the pores already filled with residual carbon due to PRBI and pyrolysis treatment. The 1st hybrid 1 h-CIP-3 sample has a lower initial porosity and due to this fact, the lower amount of residual carbon is required to fill the pores and thus increase density.

### Microhardness measurements

**Figure 8:**
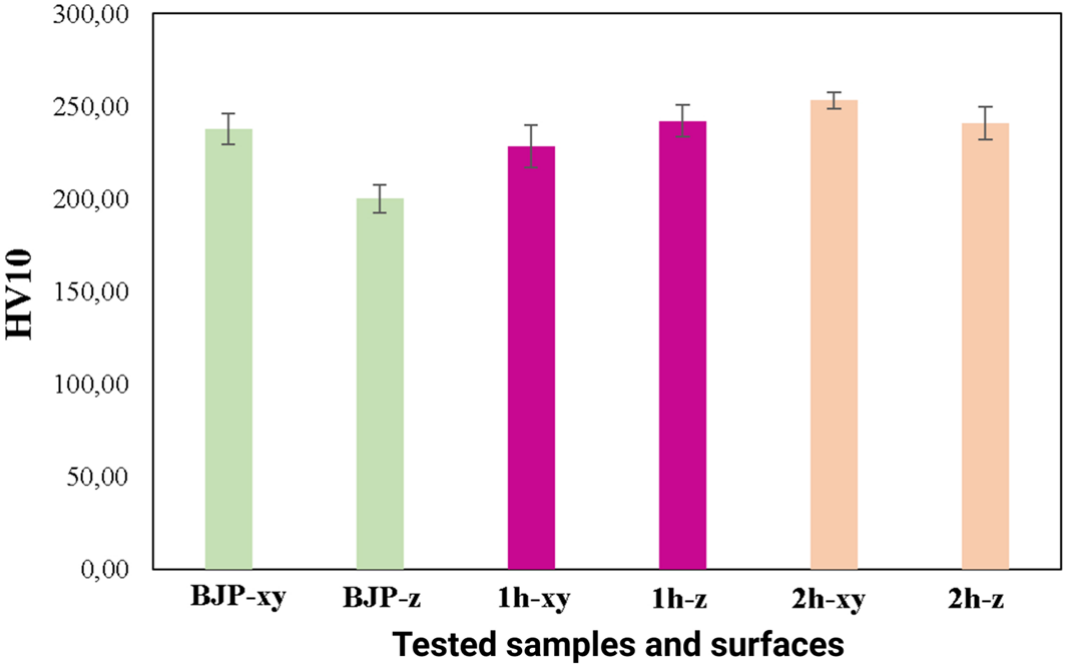
Vickers hardness HV10 of different samples groups (5 samples in each one) measured and different samples surfaces.

Microhardness measurements of green samples revealed a homogeneous microhardness of about of HV10 233.8 MPa (see Fig. 8).

An impregnation frequency of five times followed by pyrolysis resulted in a density improvement from 0.94 to 1.31 g/cm^3^ without causing any microhardness increase. This result may be explained by lower hardness of the impregnated carbon comparable to the as-printed graphite. Samples produced by the 1st hybrid method demonstrated higher microhardness than those undergoing only impregnation/pyrolysis treatment, but their homogeneity from the point of view of microhardness remained low. Obviously, additional heat treatment is required to improve the homogeneity of CIPed samples.

### Complex-shape part fabrication by the hybrid method

**Figure 9:**
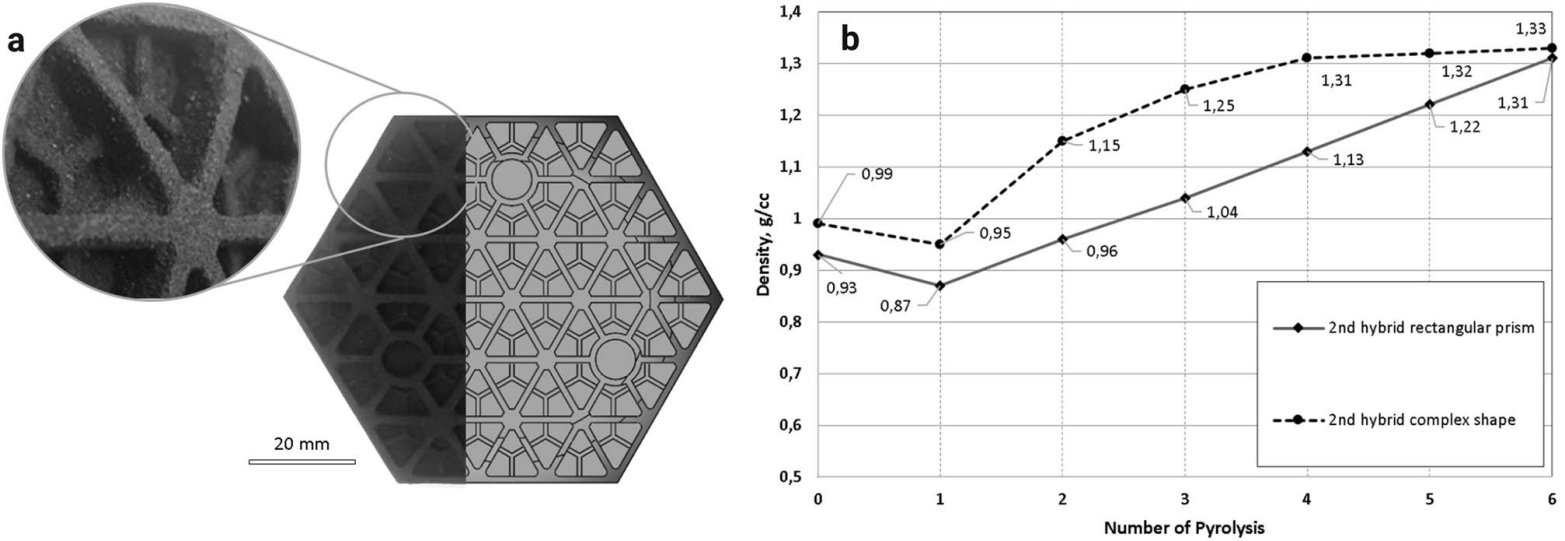
(**a**) Overview of graphite BJP complex structures densified through PRBI and pyrolysis cycles; (**b**) dependence of the hybrid complex-shape sample’s density on the number of pyrolysis. The 2nd up to 6th pyrolysis was applied after PRBI.

We successfully printed a complex-shape mirror-like component to demonstrate the applicability of the proposed hybrid techniques while quantifying the differences in the printed density between simple and complex shapes. We processed the complex-shape part by the 2nd hybrid method. Figure 9 shows that this part densified faster than the simple shape rectangular prisms. Although the ultimate density was the same, the densification rate differed which may be explained from the larger surface area of impregnation.

## Summary

This study introduces two hybrid processes integrating an additive manufacturing technique with post-processing treatments namely (i) Binder Jetting Printing (BJP) + Cold Isostatic Pressing (CIP) + cycle and (ii) BJP + cycle where cycle refers to a sequence of Impregnation—Drying—Pyrolysis shown by simple representation as below:

(i) BJP → CIP → Impregnation → Pyrolysis
(ii) BJP → Impregnation → Pyrolysis

After several cycles of impregnation and pyrolysis for debinded printed high-porous specimens, the density of the printed graphite improved by up to 96%. Moreover, applying CIP treatment to as-printed samples reduces the cycles of impregnation and pyrolysis while improving the density by almost up to 100%.

The dependence of geometry and propensity of densification and the homogeneity of carbonisation are promising gaps for further research.

## Conclusions

This novel research demonstrates for the first time the possibility of manufacturing high-density graphite structures using the Binder Jetting process. Enroute to this research effort, graphite parts with near theoretical density (without waste) were fabricated. This research shows that the proposed post-processing sequence provides a significant reduction of porosity in the graphite structures fabricated by BJP. Two manufacturing routes were explored and both routes showed benefits as described below:

- The 1st hybrid technological chain involving an intermittent step of cold isostatic pressing (CIP) was found to be preferable since it provides higher density and helps achieve complex geometries but without thin and too small elements. These results were achieved due to high densification effect of isostatic pressing in closely porous bodies.
- The 2nd hybrid chain with 5-cycles of impregnation and pyrolysis is preferable to produce complex geometrical structures. This result is achieved due to high liquid permeability of BJ printed green bodies with controlled open porosity and, therefore, due to the high potential of porosity closure in complicated-shaped parts.
- Overall, the introduction of CIP after BJP helped to reduce defects/porosity and also aids in reducing the number of cycles required to achieve the same fabrication performance which would otherwise be achieved with a much larger number of impregnation and pyrolysis cycles.

## Data Availability

Data can be accessed from 10.17862/cranfield.rd.13340945.
