# Author Correction: Novel hybrid method to additively manufacture denser graphite structures using Binder Jetting

**DOI:** 10.1038/s41598-022-05818-3

**Published:** 2022-01-25

**Authors:** Vladimir Popov, Alexander Fleisher, Gary Muller‑Kamskii, Shaul Avraham, Andrei Shishkin, Alexander Katz‑Demyanetz, Nahum Travitzky, Yair Yacobi, Saurav Goel

**Affiliations:** 1 Israel Institute of Metals, Technion, Israel Institute of Technology, 3200003 Haifa, Israel; 2 Institute of New Materials and Technologies, Ural Federal University, Ekaterinburg, Russia; 3 NRCN, Nuclear Research Center Negev, P.O. Box 9001, 8419001 Beer-Sheva, Israel; 4 Rudolfs Cimdins Riga Biomaterials Innovations and Development Centre of RTU, Institute of General Chemical Engineering, Faculty of Materials Science and Applied Chemistry, Riga Technical University, Riga, 1007 Latvia; 5 Maritime Transport Department, Latvian Maritime Academy, Riga, 1016 Latvia; 6 Department of Materials Science and Engineering, Institute of Glass and Ceramics, University of Erlangen-Nuremberg, 91058 Erlangen, Germany; 7 School of Engineering, London South Bank University, London, SE10AA UK; 8 School of Aerospace, Transport and Manufacturing, Cranfield University, Cranfield, MK43 0AL UK; 9 Department of Mechanical Engineering, Shiv Nadar University, Gautam Budh Nagar, 201314 India

Correction to: *Scientific Reports* 10.1038/s41598-021-81861-w, published online 28 January 2021

The original version of this Article contained errors.

Firstly, Shaul Avraham and Yair Yacobi were omitted from the author list in the original version of this Article.

The Author Contributions section now reads:

V.P., G.M.-K. were involved in the experimental protocols, including the specimens’ fabrication, preparation and characterization of them. A.K.-D. made the XRD examination and together with A.S., contributed to results interpretation. S. A., Y. Y. contributed to the research formulation, provided scientific advice and supervision. N.T., A.F., and S.G. provided scientific advice and supervision. All authors have contributed to the writing of this article.

Secondly, Affiliation 2 were incorrectly given as ‘Heat Treatment and Physics of Metals Department, Ural Federal University, Ekaterinburg, Russia.’ The correct affiliation is listed below:

Institute of New Materials and Technologies, Ural Federal University, Ekaterinburg, Russia

Thirdly, the Affiliations given for Vladimir Popov were incomplete. The correct affiliations are listed below:

Israel Institute of Metals, Technion, Israel Institute of Technology, 3200003, Haifa, Israel

Institute of New Materials and Technologies, Ural Federal University, Ekaterinburg, Russia

Fourthly, the incorrect email address for Vladimir Popov was quoted. Correspondence and requests for materials should be addressed to vvp0604@gmail.com.

Lastly, the Acknowledgements section was incomplete.

“This work performed in the frame of ITHACA, the COST INNOVATORS' GRANT, supported by COST (European Cooperation in Science and Technology). All authors greatly acknowledge the financial support provided by the UKRI via Grants No. EP/L016567/1, EP/S013652/1, EP/S036180/1, EP/T001100/1 and EP/T024607/1, Royal Academy of Engineering via Grants No. IAPP18-19\295, TSP1332 and EXPP2021\1\277, EU Cost Action (CA15102, CA18125, CA18224 and CA16235) and Newton Fellowship award from the Royal Society (NIF\R1\191571). SG is particularly thankful to European Regional Development Funds (ERDF) sponsored A2i project at LSBU that have catalysed several industrial partnerships.”

now reads:

“This research was funded by the NRCN. This work performed in the frame of ITHACA, the COST INNOVATORS' GRANT, supported by COST (European Cooperation in Science and Technology). All authors greatly acknowledge the financial support provided by the UKRI via Grants No. EP/L016567/1, EP/S013652/1, EP/S036180/1, EP/T001100/1 and EP/T024607/1, Royal Academy of Engineering via Grants No. IAPP18-19\295, TSP1332 and EXPP2021\1\277, EU Cost Action (CA15102, CA18125, CA18224 and CA16235) and Newton Fellowship award from the Royal Society (NIF\R1\191571). SG is particularly thankful to European Regional Development Funds (ERDF) sponsored A2i project at LSBU that have catalysed several industrial partnerships.”

The original Article has been corrected.

